# Recent advances in Lynch syndrome

**DOI:** 10.1186/s40164-021-00231-4

**Published:** 2021-06-12

**Authors:** Xi Li, Guodong Liu, Wei Wu

**Affiliations:** 1 Department of Geriatric Surgery, Xiangya Hospital, Central South University, Changsha, 410008 China; 2 National Clinical Research Center for Geriatric Disorders, Xiangya Hospital, Central South University, Changsha, 410008 China; 3 Department of General Surgery, Xiangya Hospital, Central South University, Changsha, 410008 China

**Keywords:** Lynch syndrome, DNA mismatch repair, Colorectal cancer, Endometrial cancer, Immunotherapy

## Abstract

Lynch syndrome is one of the most common hereditary cancer syndromes and is characterized by the development of many cancers, such as colorectal cancer (CRC), endometrial cancer, ovarian cancer, stomach cancer and many other cancers. Lynch syndrome is caused by pathogenic germline variants in one of four DNA mismatch repair genes (MLH1, MSH2, MSH6, or PMS2) or by an EPCAM deletion. The MLH1 variant is correlated with the highest risk of CRC, while the MSH2 variant is correlated with the highest risk of other cancers. CRC is the most common cancer type that develops in individuals with Lynch syndrome, followed by endometrial cancer. Recent advances have been made to help us further understand the molecular pathogenesis of this disease and help improve diagnostic testing efficiency and surveillance strategies. Moreover, recent advances in immunotherapy provided by clinical trials also provide clinicians with more chances to better treat Lynch syndrome. This study aims to review many advances in the molecular genetics, clinical features, diagnosis, surveillance and treatment of Lynch syndrome.

## Background

Lynch syndrome (LS), which was previously called hereditary nonpolyposis colorectal cancer (HNPCC), is one of the most common hereditary cancer syndromes and often leads to various types of tumors at a young age. In 2009, Dr. Henry T. Lynch defined germline mutations in the DNA mismatch repair (MMR) system as Lynch syndrome [1]. The population defined by the original HNPCC definition was divided into two parts: one is Lynch syndrome, which refers to the population with pathogenic mutations in the germline MMR gene, and the remaining colorectal cancers with a family history but no MMR germline mutations were newly named "familial colorectal cancer type X". The estimated prevalence of LS in the general population ranges from 1/1000 to 1/250 [2–5] and accounts for 1–4% of all CRC cases [6–8]. LS develops at early ages in individuals with various cancers, among which CRC and endometrial cancer (EC) rank as the top two involved cancers among other associated cancers, including ovarian, stomach, small bowel, urinary tract, biliary tract, brain, skin (sebaceous adenomas, sebaceous carcinomas, and keratoacanthomas), pancreatic, and prostate cancers [9, 10]. Some studies have demonstrated that other cancers, such as breast cancer, sarcoma, and adrenocortical carcinoma, occur in individuals with Lynch syndrome, but until now, the data have been far from sufficient to define the correlation between the risk of these cancers and Lynch syndrome [11–15].

## Molecular genetics

Lynch syndrome is caused by pathogenic germline variants in four DNA mismatch repair (MMR) genes (MLH1, MSH2, MSH6, or PMS2) or by an EPCAM deletion; thus, the screening of such variants and EPCAM deletion analysis are often recommended for the diagnosis of LS [16–19]. The function of DNA MMR is to maintain genomic stability, and the dysfunction of DNA MMR could lead to alterations in the repetitive sequence number of microsatellites, which is defined as microsatellite instability (MSI). Commonly, high frequency of MSI (MSI-H) is shown in tumors developed in LS individuals with variants in MMR genes. Usually, individuals with pathogenic variants of each gene often have a risk of developing different cancers. The cumulative cancer incidences of MLH1, MSH2, MSH6, or PMS2 variant carriers up to age 75 are as follows: MLH1: 81% (females), 71.4% (males); MSH2: 84.3% (females), 75.2% (males); MSH6: 61.8% (females), 41.7% (males); and PMS2: 34.1% (both sexes). From age 50, the cumulative cancer incidences of MLH1 or MSH2 variant carriers increase rapidly, and for MSH6 or PMS2 variant carriers, the cumulative cancer incidences often increase rapidly from age 60 [20].

A study based on data from the United States, Canada, and Australia via the Colon Cancer Family Registry (CCFR) collected data from 5744 CRC patients and 37,634 first-degree relatives and showed that the carrier frequency of pathogenic germline variants in the four MMR genes within the general population was 0.051% (1:1946) for MLH1 mutations, 0.035% (1:2841) for MSH2 mutations, 0.132% (1:758) for MSH6 mutations, and 0.140% (1:714) for PMS2 mutations, resulting in an aggregate carrier [21].

### MLH1

The MLH1 variant is correlated with the highest risk of developing CRC [21, 22], with a cumulative cancer incidence ranging from 0% (age 30) to 48.3% (age 75) in females and from 4.5% (age 30) to 57.1% (age 75) in males [20]. A study showed that a significant proportion of patients with Bethesda criteria who have loss of MLH1 protein expression in their tumors and do not have an MLH1 pathogenic germline mutation display constitutional MLH1 methylation as the mechanism of Lynch syndrome, especially patients with CRC diagnosed before age 50, with multiple Lynch syndrome-associated tumors and no significant family history of early-onset disease [23]. Additionally, MLH1 is associated with a high risk of EC, with a cumulative cancer incidence of 37% among individuals aged 75 [20].

### MSH2

The MSH2 variant is correlated with the highest risk of developing many cancers, except CRC, with cumulative cancer incidences of 48.9% for EC, 17.4% for ovarian cancer, 18.7% (females) and 17.6% (males) for ureter and kidney cancers, and 23.8% for prostate cancer [20]. Moreover, the MSH2 variant is correlated with the second highest risk of CRC, only slightly lower than MLH1, with cumulative cancer incidences of 46.6% (females) and 51.4% (males) among individuals aged 75 [20].

### MSH6

The cumulative incidences of overall cancers for MSH6 pathogenic variant carriers increase rapidly from age 50, especially for those older than 60, increasing from 18.2% (age 50) to 41.6% (age 60) in females and from 14% (age 50) to 25% (age 60) in males [20]. At the same time, cancers in individuals with the MSH6 pathogenic variant occur later than in those with the MLH1 or MSH2 pathogenic variant, with cumulative cancer incidences of 2.7% (females) and 6.3% (males) among individuals aged 40 [20]. Moreover, female MSH6 pathogenic variant carriers are at high risk of endometrial cancer compared with caners in other organs, and the CRC risk associated with the MSH6 variant is lower than that in MLH1 and MSH2 variant carriers [20].

### PMS2

The cumulative incidences of overall cancers for PMS2 pathogenic variant carriers are the lowest among the four pathogenic germline variants, with cumulative cancer incidences of 34.1% at the age of 75 [20]. Studies have reported that although PMS2 pathogenic variant carriers may not increase the tumorigenesis of CRC, the lack of PMS2 protein can promote the progression of MMR mature adenoma to CRC [24], which indicates that surveillance and polypectomy may be effective strategies to prevent CRC in these pathogenic variant carriers.

### EPCAM

EPCAM is highly expressed in epithelial tissues and tumors. Studies have shown that 3ʹ end EPCAM deletion is a recurrent cause of LS, and these truncating EPCAM deletions cause allele-specific epigenetic silencing of the neighboring DNA mismatch repair gene MSH2 and subsequent hypermethylation of its CpG island promoter in tissues expressing EPCAM [25–27]. The incidence of EPCAM deletion varies among populations and was shown to account for at least 1–3% of the explained Lynch syndrome families. Therefore, deletion analysis of EPCAM is appropriate for the diagnosis of Lynch syndrome. Individuals with deletions of EPCAM have a high risk of developing CRC. Unlike the pathogenic mutation sof MSH2, EPCAM deletion usually rarely causes extragastrointestinal tumors [28].

## Clinical features

### Colorectal cancer

CRC is the most common cancer type that develops in individuals with Lynch syndrome. According to studies, individuals with MLH1 and MSH2 pathogenic variants have higher cumulative incidences of CRC than those with MSH6 and PMS2 pathogenic variants, with incidences of 48.3% (females) and 57.1% (males) in those aged 75 with the MLH1 pathogenic variant and 46.6% (females) and 51.4% (males) in those with the MSH2 pathogenic variant [20], which is much higher than that in the general population (2% by age 74) [29, 30]. At the same time, the data also showed that the age of onset of MSH6 and PMS2 pathogenic variant carriers was later than that of MLH1 and MSH2 pathogenic variant carriers, with ages of 40–75 and 70–75, respectively [11, 20], which indicates the importance of early surveillance for individuals with MLH1 and MSH2 pathogenic variants.

### Endometrial cancer

Endometrial cancer ranks as the second most common cancer type that develops in individuals with Lynch syndrome [20, 22, 31]. Many studies have reported that the MSH2 pathogenic variant is associated with the highest risk of endometrial cancer, with a cumulative incidence of 48.9% in those aged 75 [20]. Individuals with MSH6 and MLH1 pathogenic variants have the second and third highest risks of developing endometrial cancer, with cumulative incidences of 41.1% and 37%, respectively, in those aged 75 [20]. Although the incidence may range according to different studies, all the studies showed the same tendency. For those with the PMS2 pathogenic variant, endometrial cancer may develop later, and the cancer risk is often lower than that of individuals with the other three pathogenic variants, with a cumulative incidence of 0 before age 50 and 9.3%-12.8% at age 60–70 [20].

### Ovarian cancer

The risk of ovarian cancer developing in the four gene pathogenic variant carriers is obviously lower than that of colorectal cancer and endometrial cancer. The risk of ovarian cancer in Lynch syndrome is mainly associated with MLH1, MSH2 and MSH6 pathogenic variants, and the MSH2 pathogenic variant showed the highest risk among the four gene variants, with a cumulative incidence ranging from 10.8 to 17.4% by age 75 [20]. Ovarian cancer does not occur in Lynch syndrome before the age of 40, and the PMS2 pathogenic variant only shows a 3% incidence at the age of 60–75 [20].

### Breast cancer

Usually, the risk of the general population developing breast cancer by age 74 is 5% [29, 30], but for individuals with Lynch syndrome, by age 70, the risk increases to 11%, 13%, 11%, and 8% with the MLH1, MSH2, MSH6, and PMS2 pathogenic variants, respectively [20]. In another study, the risk of developing breast cancer in individuals with Lynch syndrome with the MLH1, MSH2, MSH6, and PMS2 pathogenic variants increased to 12.3%, 14.6%, 13.7%, and 15.2%, respectively, by age 75 [20].

## Adrenal cortical carcinoma (ACC)

ACC is an uncommon endocrine cancer with an incidence of 0.5–2/1,000,000 per year [32]. Most ACCs diagnosed during childhood or adolescence are related to hereditary syndromes, such as Li-Fraumeni syndrome (main cause), multiple endocrine neoplasia type 1 (MEN-1) and LS. According to a retrospective cohort study from the Catalan Institute of Oncology Hereditary Cancer Registry that included 634 individuals (347 women and 287 men) from 220 families diagnosed with LS between 1999 and 2018, the incidence of developing nonsecreting ACCs was 0.47% among all of the patients included (3/634), which was 70-fold higher than that in the general population for this study period (1999–2018). Moreover, all 3 ACC patients carried a germline mutation in the MSH2 gene, and 1.7% of all patients had an MSH2 mutation [33]. Genetic evaluation for LS should be considered in all patients with ACC with a personal or family history of LS-associated tumors.

## Variants of Lynch syndrome

### Muir-Torre syndrome

Muir-Torre syndrome is a phenotypic variant of Lynch syndrome that is characterized by the presence of skin sebaceous neoplasms and one or more visceral cancers. To date, many types of skin tumors have been reported in Lynch syndrome, including sebaceous carcinoma, keratoacanthoma, sebaceous adenoma, sebaceous epithelioma and cutaneous squamous cell carcinoma [34].

### Constitutional mismatch repair deficiency (CMMRD) syndrome

CMMRD, also called biallelic MMR deficiency, is caused by homozygous or biallelic germline variants in MMR genes. Brain tumors, CRC and hematological tumors are the most common tumor types that develop in CMMRD. Unlike LS, PMS2 is the most common MMR gene observed in CMMRD [35].

## Diagnosis

Usually, patients who meet the Amsterdam criteria II (Table 1) [36] or revised Bethesda guidelines (Table 2) [31] are often recommended for further testing of LS; however, this is not enough to screen all LS cases because some studies reported that more than ¼ of cases would be missed [6]. Ninety percent of CRC patients with LS show high-frequency MSI (MSI-H) or abnormalities in immunohistochemistry (IHC) [2, 37], so MSI testing or IHC is recommended for the screening of patients with CRC [38]. IHC can be performed to evaluate the protein expression of MLH1, MSH2, MSH6, and PMS2, which can serve as a supplement to MSI testing [8]. In addition, a systematic review showed that there was no significant test accuracy difference between IHC- and MSI-based strategies for EC testing [39]. MMR gene testing is recommended for patients with MMR-D (deficient MMR protein expression, an indicator of epigenetic hypermethylation of the MLH1 promoter region in most instances). Moreover, MMR-D CRC can also serve as a predictive, prognostic, and therapeutic marker for LS [40]. BRAF V600E testing can be used to exclude sporadic MSI-H CRC [2, 41, 42] because the BRAF V600E somatic variant is present in approximately 40% of sporadic MSI-H CRC cases but rarely in LS [43, 44]. BRAF pathogenic variants are not common in sporadic endometrial cancers; thus, BRAF testing is not helpful in distinguishing endometrial cancers that are sporadic from those that are Lynch syndrome related [45]. Although EPCAM is not a mismatch repair gene, recurrent germline deletions of the 3' region result in the silencing of the adjacent downstream MSH2 gene by hypermethylation, and deletion analysis of EPCAM is appropriate for the diagnosis of Lynch syndrome.

**Table 1:** Amsterdam criteria II

At least three relatives must have a Lynch syndrome-associated cancer (colorectal, endometrial, small bowel, ureter, or renal pelvic cancer); all of the following criteria should be met:
1. One must be a first-degree relative of the other two
2. At least two successive generations must be affected
3. At least one should have been diagnosed before the age of 50 years
4. Familial adenomatous polyposis should be excluded
5. Tumor diagnosis should be confirmed by histopathological examination

**Table 2:** Revised Bethesda guidelines for colorectal cancers for microsatellite instability testing

Tumors from patients with colorectal cancer (CRC) should be tested for MSI in the following situations:
1. CRC diagnosed in a patient less than 50 years
2. Presence of synchronous, metachronous colorectal, or other Lynch syndrome (LS)-associated tumors, regardless of age
3. CRC with MSI-H phenotype diagnosed in a patient less than 60 years
4. CRC diagnosed in a patient with one or more first-degree relatives with an LS-associated tumor, with one of the cancers being diagnosed under the age of 50 years
5. CRC diagnosed in two or more first- or second-degree relatives with LS-associated tumors, regardless of age

In addition, due to the heavy costs and the fact that universal screening cannot cover each individual with LS, family history recording and genetic testing of relatives are still important supplementary methods for the diagnosis of LS. For individuals with no tumor or those unable to undergo tumor assessment of microsatellite instability but have a family history of LS, clinical prediction models, such as PREMM (http://premm.dfci.harvard.edu/), are recommended [46]. Many types of variants have been reported to be related to LS [47], and multigene panel testing is recommended to detect pathogenic variants due to its high detection rate and cost-effectiveness. Moreover, multigene panel testing can also clarify the relations of genotype-phenotype [48].

However, by implementing the next-generation sequencing (NGS) method, more information can be obtained at a lower cost and in less time, thereby improving the detection efficiency [49, 50]. Advances in molecular testing and NGS technologies now allow all patients with colorectal and endometrial cancers to reliably receive screening for underlying Lynch syndrome, whereas innovations in immuno-oncology promise to continue revolutionizing the treatment of Lynch syndrome-associated cancers.

## Surveillance

### CRC

The risk of developing CRC before age 25 is very low, which has been supported by many studies [51–55], and the surveillance of CRC is recommended beginning between age 20 and age 25. Periodic examination by colonoscopy has been suggested to be a useful method to detect CRC at an earlier stage, and some studies have even shown that surveillance by colonoscopy regularly could lead to a 63% reduction in CRC and can also significantly reduce the mortality associated with CRC [56, 57]. Studies have shown that the progression from colon adenoma to cancer in patients with LS is faster than that in the general population, and Dukes A and B tumors are often detected within an interval of 2 years [58, 59], so conducting colonoscopy every 1–2 years is recommended. For individuals with a CRC family history without evidence of LS, colonoscopy should be conducted every 3–5 years, beginning at 5–10 years before the first diagnosis of CRC or over the age of 45 years.

Increasing evidence has shown that obesity, a high-fat diet, smoking and type 2 diabetes could increase the risk of developing CRC in individuals with LS [60], but the intensity of the impact is the same as that in ordinary people [61]. Therefore, it is important for LS patients to be aware about these situations to reduce the risk of developing CRC.

### EC and ovarian cancer

To date, only a few studies have been conducted to study the efficiency of surveillance for endometrial cancer in families with Lynch syndrome, and more studies are needed to obtain constructive opinions. Gynecological examination, transvaginal ultrasound (TVU) and aspiration biopsy beginning at the age of 30 to 35 may help to detect precancerous lesions and early cancers [62–64]. Prophylactic hysterectomy and salpingo-oophorectomy may be useful for women with LS after menopause or for women who require surgery for CRC [65].

### Other related cancers

Other related cancers of LS include cancer of the stomach, small bowel, urinary tract, biliary tract, brain, skin, pancreas, and prostate. The risk of developing these cancers in individuals with LS is very low. For those with gastric-duodenal cancers, especially individuals with a family history of gastric cancer or those of Asian ancestry [66, 67], upper gastrointestinal endoscopy is recommended every 3–5 years beginning at the age of 30–35. The presence of Helicobacter pylori infection should be evaluated from approximately age 25 so that patients could undergo treatment as needed [68, 69]. For distal small bowel cancers, capsule endoscopy/small bowel enterography is recommended. For urinary tract cancers, microscopic hematuria identified by urine analysis and urine cytology in individuals with a family history of urothelial cancer every year beginning at the age of 30–35 may be considered. For pancreatic cancer, EUS and/or MRI/MRCP examination annually may be useful for individuals with a family history.

## Genetic counseling and pregnancy management

LS is an autosomal dominant genetic disease with pathogenic germline variants in MLH1, MSH2, MSH6, or PMS2 or an EPCAM deletion. The majority of individuals with Lynch syndrome inherited a pathogenic variant from a parent. Individuals with LS have a 50% chance of passing on the pathogenic variant to the next generation. If the pathogenic variant in the family is confirmed, prenatal testing for a pregnancy at increased risk is recommended. Cancer screening is recommended for females with LS before pregnancy. If cancer develops in females with LS during pregnancy, the options for cancer treatment and their potential implications for the fetus should be provided when counseling.

## Treatment

### Surgery for CRC

Studies have shown that individuals with LS have a risk of developing multiple CRCs at different colorectal segments, so routine total colon examination before resection of a colon tumor for these patients is necessary. At the same time, individuals with LS have a risk of developing a second CRC after resection of primary CRC [70]. Therefore, whether to conduct total colectomy or segmental resection for these patients is an issue that clinicians must consider. However, to date, no consensus on this issue has been reached. Extended colectomy for patients with colon cancer and LS is strongly recommended by the US Multi-Society Task Force on Colorectal Cancer [42], but it is only weakly recommended by the Mallorca group (a European group) [69]. Studies have shown that conducting subtotal colectomy at a young age (47 years) would lead to an increased life expectancy of up to 2.3 years [71].

### Surgery for EC and ovarian cancer

Prophylactic hysterectomy and bilateral salpingo-oophorectomy, especially during CRC surgery, have been reported to be useful for reducing the risk of EC and ovarian cancer [65], but the age of patients and their desire to have children, menopausal status, gene variant type and stage of CRC are issues that clinicians and patients must consider.

### Chemotherapy for CRC

The long-term use of aspirin (600 mg/day, at least 2 years) has been reported to significantly reduce the risk of developing CRC and extracolonic LS-associated tumors by the Colorectal Adenoma/Carcinoma Prevention Programme (CAPP2) chemoprevention trial [72].

Only a small number of CRCs respond to 5-FU, and many studies have reported resistance to commonly used chemotherapeutic agents, such as cisplatin and 5-FU. Both the in vitro predictions and the empirical observations support the conclusion that patients with LS or with the acquired form of MSI due to the methylation-induced silencing of MLH1 should not be offered adjuvant chemotherapy with a 5-FU-based regimen. Both in vitro predictions and empirical observations suggest that 5-FU-based regimens should not be recommended for patients with LS or MSI/acquired MSI due to methylation-induced MLH1 silencing.

Only a few studies have reported the efficiency of chemotherapy in patients with MSI-H or HNPCC tumors, and most of the studies found that 5-FU treatment could not improve the prognosis of these patients [73–75]. One study reported that oxaliplatin could improve prognosis in stage III colon cancer with MSI-H. More studies are still needed to recommend chemotherapy for CRC patients with LS. Another case reported that dabrafenib alone or combined with trametinib would benefit the therapy of MSI-H BRAF V600E-mutated endometrial adenocarcinoma [76].

### Immunotherapy

Immunotherapy has been proven to show prospects for tumor treatment [77–80]. Some clinical trials are being conducted to explore the efficiency of immune-based therapies for the prevention of cancers in individuals with LS [81, 82]. A clinical trial (NCT02060188) concluded that nivolumab plus ipilimumab provides a promising new treatment option for patients with dMMR/MSI-H mCRC, with an investigator-assessed ORR of 55% (95% CI, 45.2 to 63.8) and a disease control rate at ≥ 12 weeks of 80% [83]. Moreover, a recent study indicated that the degree of microsatellite instability could predict a patient’s response to anti-PD-1 immunotherapy, and high microsatellite instability was an independent predictor of longer PFS in dMMR/MSI-H CRCs [84].

## Conclusions

Many studies have been conducted to help improve our understanding of LS in the past few years, especially in the aspects of molecular genetics, clinical features, diagnosis, surveillance and treatment. At the same time, more evidence is still needed to obtain consensus on the management of LS, such as the choice of surgery methods, use of chemotherapy drugs and immunotherapy plan. Immunotherapy shows good prospects for improving the prognosis of individuals with LS.

## Data Availability

Not applicable.

## Abbreviations

ACC: Adrenal cortical carcinoma
CAPP2: Colorectal Adenoma/Carcinoma Prevention Programme
CMMRD: Constitutional mismatch repair deficiency
CRC: Colorectal cancer
EC: Endometrial cancer
HNPCC: Hereditary nonpolyposis colorectal cancer
IHC: Immunohistochemistry
LS: Lynch syndrome
MEN-1: Multiple endocrine neoplasia type 1
MMR: Mismatch repair system
MMR-D: Deficient MMR protein expression
MSI: Microsatellite instability
MSI-H: High frequency of MSI
TVU: Transvaginal ultrasound
